# Snail and Slug collaborate on EMT and tumor metastasis through miR-101-mediated EZH2 axis in oral tongue squamous cell carcinoma

**DOI:** 10.18632/oncotarget.3180

**Published:** 2015-02-09

**Authors:** Min Zheng, Ya-ping Jiang, Wei Chen, Kai-de Li, Xin Liu, Shi-yu Gao, Hao Feng, Sha-sha Wang, Jian Jiang, Xiang-rui Ma, Xiao Cen, Ya-jie Tang, Yu Chen, Yun-feng Lin, Ya-ling Tang, Xin-hua Liang

**Affiliations:** ^1^ State Key Laboratory of Oral Diseases West China Hospital of Stomatology (Sichuan University), Chengdu Sichuan 610041, People's Republic of China; ^2^ Department of Stomatology, Zhoushan Hospital, Zhoushan, Zhejiang 316000, People's Republic of China; ^3^ Department of Oral and Maxillofacial Surgery, Tianjin Stomatological Hospital, Tianjin 300041, People's Republic of China; ^4^ Key Laboratory of Fermentation Engineering (Ministry of Education), Hubei University of Technology, Wuhan 430068, People's Republic of China; ^5^ Department of Oral Pathology, West China Hospital of Stomatology (Sichuan University), Chengdu Sichuan 610041, People's Republic of China; ^6^ Department of Oral and Maxillofacial Surgery, West China Hospital of Stomatology (Sichuan University), Chengdu Sichuan 610041, People's Republic of China

**Keywords:** Oral tongue squamous cell carcinoma (OTSCC), epithelial-mesenchymal transition (EMT), miR-101, Snail, Slug

## Abstract

microRNAs(miRNAs) can regulate epithelial-mesenchymal transition (EMT) through transcription factors, however, little is known whether EMT transcription factors can modulate miRNAs and further induce EMT and cancer metastasis. Here we show that overexpression of Snail and Slug leads to a mesenchymal phenotype and morphology and enhances cell invasion along with stem cell properties in squamous cell carcinoma of oral tongue (OTSCC) cells. Repression of miR-101 expression by Snail and Slug is essential for Snail/Slug-induced malignant phenotypes. The suppression of miR-101 subsequently activates EZH2, the sole histone methyltransferase, inducing EMT, migration and invasion of OTSCC cells. Importantly, co-overexpression of Slug and Snail correlates with poor survival and elevated EZH2 expression in two independent patient cohorts of OTSCC specimens. These findings defined a Snail and Slug/miR-101/EZH2 pathway as a novel regulatory axis of EMT-mediated-microRNA signaling.

## INTRODUCTION

Squamous cell carcinoma of the head and neck (HNSCC) is the sixth most prevalent cancer in the world [1]. Despite its significance and the enormous knowledge accumulated in the past decades on the mechanisms of HNSCC generation and progression, very little is still known about the molecular mechanisms governing metastatic dissemination [2]. Epithelial-mesenchymal transitions (EMT), interconverting epithelial cell types into cells with mesenchymal attributes, has been shown to play a pivotal role in various steps of tumor metastatic cascades, including HNSCC [3, 4]. Snail (Snail1) and Slug (Snail2), the Snail family transcription factors, are strong E-cadherin repressors and major EMT inducers, express in breast cancers, ovarian cancers, and colon cancers, and involve in tumor metastasis and recurrence [5–8]. Importantly, Snail and Slug have been reported to collaborate on primary tumor growth and specifically contribute to site-specific metastasis of mouse skin carcinoma cell lines [9]. Thus, a comprehensive study demonstrating the interplay between Snail and Slug will provide essential information for the metastasis molecular and prognosis prediction in squamous cell carcinoma of oral tongue (OTSCC), which is one of the most common forms of HNSCC.

Recent work has demonstrated MicroRNAs (miRNAs) play critical roles in tumor EMT and metastasis. Members of the miR-200 family are well-established EMT repressors through direct targeting of ZEB1 and ZEB2 [10, 11]. miR-182 and miR-203 induce mesenchymal to epithelial transition (MET) features and growth factor independent growth via repressing SNAI2 in prostate cells [12]. MiR-300 may negatively regulate EMT by direct targeting Twist and therefore inhibit the invasion and metastasis of HNSCC [13]. These data showed that some miRNAs can regulate the expression of certain genes, such as EMT transcription factors [14, 15]. However, little is known whether EMT transcription factors can mediate miRNAs and further induce EMT and metastasis to date.

In our previous study, we described an important role for Snail, Slug and c-kit in the invasion and metastasis of salivary gland adenoid cystic carcinoma [16–18], as well as TWIST2 and SNIP1, EMT another transcription factors, in the metastasis and prognosis of OTSCC [19]. Now, we have investigated the interplay between Snail and Slug in tumor metastasis and prognosis in OTSCC clinical specimens and cell lines targeting each factor by stable RNAi, screened their miRNAs mediated by Snai1 and Slug using microRNA microarray, and analyzed their effect in the migration and invasion behavior. Our results indicated that Snail and Slug collaborate on *in vivo* and *in vitro* the invasion and metastasis, and EMT-like programs in OTSCC, and miRNA-101 (miR-101) was mediated in this process. We further showed that repression of miR-101 caused the invasion, migration and EMT of OTSCC cells through an increase in the level of enhancer of zeste homologue 2 (EZH2), the sole histone methyltransferase. Together, the present work provides the first evidence for the coordination of Snail and Slug in silencing tumor-suppressive miRNAs during OTSCC malignant progression.

## RESULTS

### Co-overexpression of Snail and Slug correlated with the metastasis and poor prognosis of OTSCC patients

**Figure 1:**
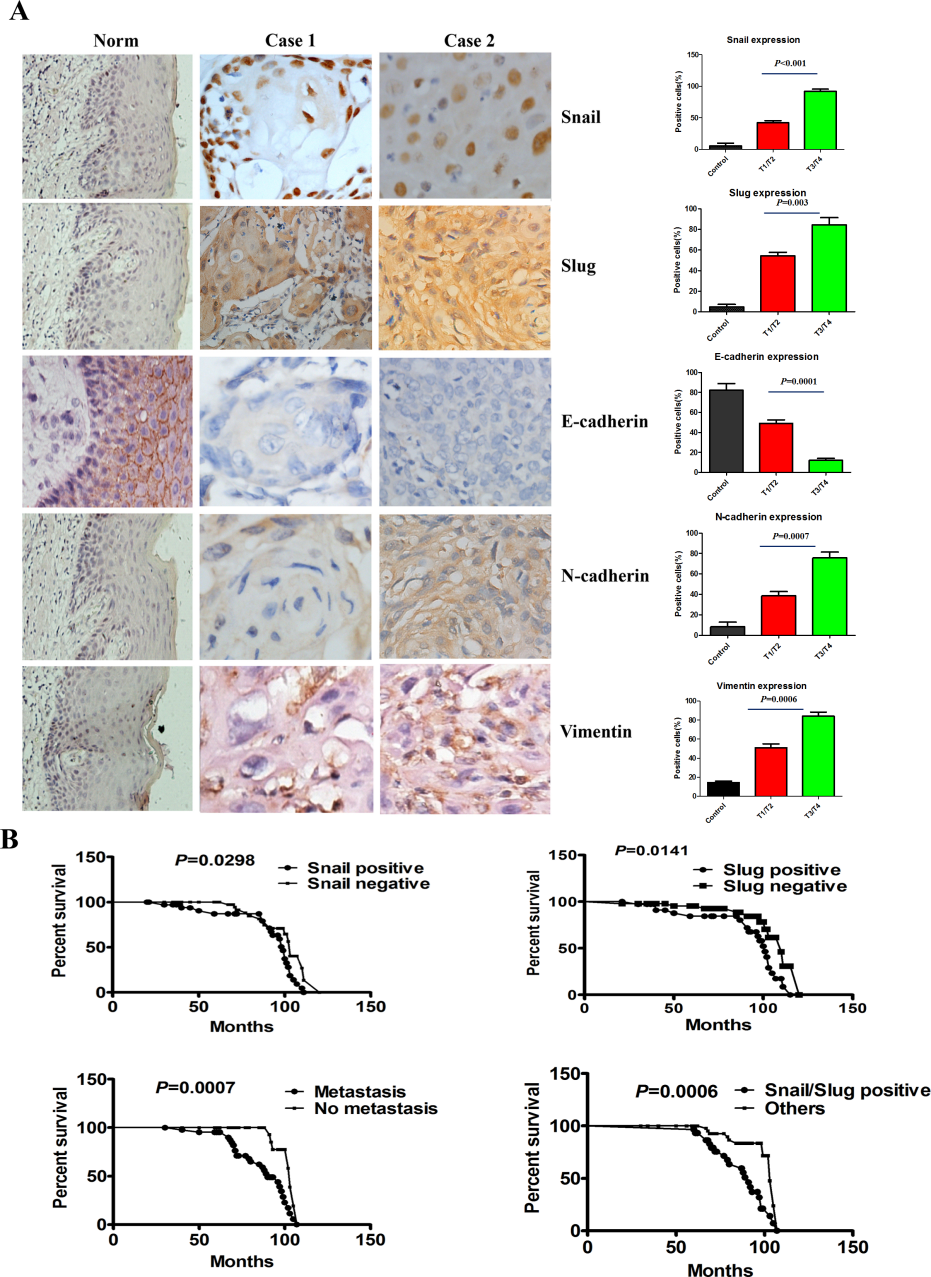
Snail and Slug expression was associated with the T classification, metastasis and poor prognosis of human OTSCC samples **(A)** Representative tissue sections (left) and quantification (right) stained for Snail, Slug, E-cadherin, N-cadherin and Vimentin by immunohistochemistry in case 1 (T1N0M0), case 2 (T3N0M0) of OTSCC samples and normal tongue mucous. Scale bar, 50 mm. **(B)** Kaplan–Meier survival analysis in patients with OTSCC. Co-overexpression of Snail and Slug in OTSCC was associated with a shorter overall survival rate, compared with the group with either of Snail and Slug overexpression and neither of them overexpression.

To evaluate the clinical relevance of Snail and Slug expression in human OTSCC, we carried out immunohistochemistry staining of Snail and Slug in 89 human OTSCC samples representing different T classification and 10 normal human tongue mucous. Representative immunohistochemical images were shown in Figure 1A. We found that the nuclear staining of Snail was detected in 36 of 89 (40.45%) OTSCC specimens, and the cytoplasm and nuclear staining of Slug were detected in 39 of 89 (43.82%). Co-overexpression of Snail and Slug was observed in 30 (33.71%) cases, either of Snail and Slug was found in 14 (15.73%) cases, and neither of them was observed in 45 (50.56%) patients with OTSCC. Snail or Slug or two protein co-expressions was significantly associated with T classification, pathological grade, and lymph node metastasis (Table 1). Moreover, overall survival curves of all patients were computed with the Kaplan–Meier method and compared between groups by using the log-rank test. The patients with positive Snail or Slug or metastasis had a poorer prognosis (a lower survival rate) than those with negative (*p* = 0.0298, *p* = 0.0141, *p* = 0.0007, respectively, Figure 1B). And a significantly worse overall survival rate patients who had both of Snail and Slug overexpression had been revealed compared with those who had one of proteins overexpression and none protein expression (*p* = 0.0006, Figure 1B).

**Table 1:** Clinicopathological features of OTSCC patients and their association with Snail and Slug expression (*n* = 89)

Clinicopatholog-ical features	*n*	Snail expression		*P* Value	Slug expression		*P* Value	Snail/Slug co-expression		*P* Value
		Positive (*n* = 36)	Negative (*n* = 53)		Positive (*n* = 39)	Negative (*n* = 50)		Both positive (*n* = 30)	Others (*n* = 59)	
**Age**	89			0.640			0.905			1.000
< = 60	53	23	30		24	29		18	35	
> 60	36	13	23		15	21		12	24	
**Gender**	89			0.466			0.659			0.371
Female	40	14	26		16	24		11	29	
Male	49	22	27		23	26		19	30	
**T classification**	89			0.007			0.001			< 0.001
T1/T2	51	14	37		14	37		9	42	
T3/T4	38	22	16		25	13		21	17	
**Pathological grade**	89			< 0.001			< 0.001			< 0.001
Well	54	6	48		12	42		5	49	
Moderate +Poorly	35	30	5		27	8		25	10	
**Lymph node metastasis**	89			< 0.001			0.006			< 0.001
Yes	37	25	12		23	14		22	15	
No	52	11	41		16	36		8	44	
**Local regional recurrence**	89			0.011			0.113			0.009
Yes	34	20	14		19	15		19	15	
No	55	16	39		20	35		11	34	

Then, co-expression of Snail and Slug, Snail or Slug expression as well as patients’ gender, age, T classification, pathologic grade, local regional recurrence, and lymph node metastasis were included in the univariate and multivariate analysis by Cox's proportional hazards regression model. The univariate analysis showed that patients’ T classification, pathologic grade, local regional recurrence, lymph node metastasis, Snail or Slug expression and co-overexpression of Snail and Slug were significantly associated with patient overall and disease-free survival (*p* < 0.05, Supplementary Table S1). All the variables, which showed *p* < 0.05 by the univariate analyses, were used for multivariate analyses. Multivariate analysis using the Cox's proportional hazards model revealed that local regional recurrence and co-overexpression of Snail and Slug pattern were independent and significant prognostic factors in overall survival of all patients (*p* = 0.004, *p* = 0.012, respectively, Supplementary Table S2), and local regional recurrence and lymph node metastasis, and co-overexpression of Snail and Slug pattern were independent and significant prognostic factors in disease-free survival of all patients (*p* < 0.001, *p* = 0.014, *p* < 0.001, respectively, Supplementary Table S2). These results suggest that Snail and Slug co-expression rather than any individual expression was the independent determinant of OTSCC prognosis. The results of IHC analysis for Snail and Slug co-expression were validated by real-time RT-PCR in representative OTSCC fresh samples (Data no shown). These observations implicate the potential usefulness of Snail and Slug co-overexpression as a novel prognostic molecular marker for OTSCC.

Simultaneously, the OTSCC specimens were immunostained for several EMT-related proteins (E-cadherin, N-cadherin and Vimentin) that are potentially relevant to tumor EMT (Figure 1A). Co-overexpression of Snail and Slug was found to be significantly associated with lower expression of E-cadherin (*p* = 0.009), higher expression of N-cadherin (*p* = 0.004), and higher expression of Vimentin (*p* = 0.002, Supplementary Table S3). These showed that Snail^+^/Slug^+^ OTSCC associated with tumor EMT.

### Co-overexpression of Snail and Slug synergistically contributed to the invasion and migration, EMT of OTSCC cells and generated stem cell-like cells

**Figure 2:**
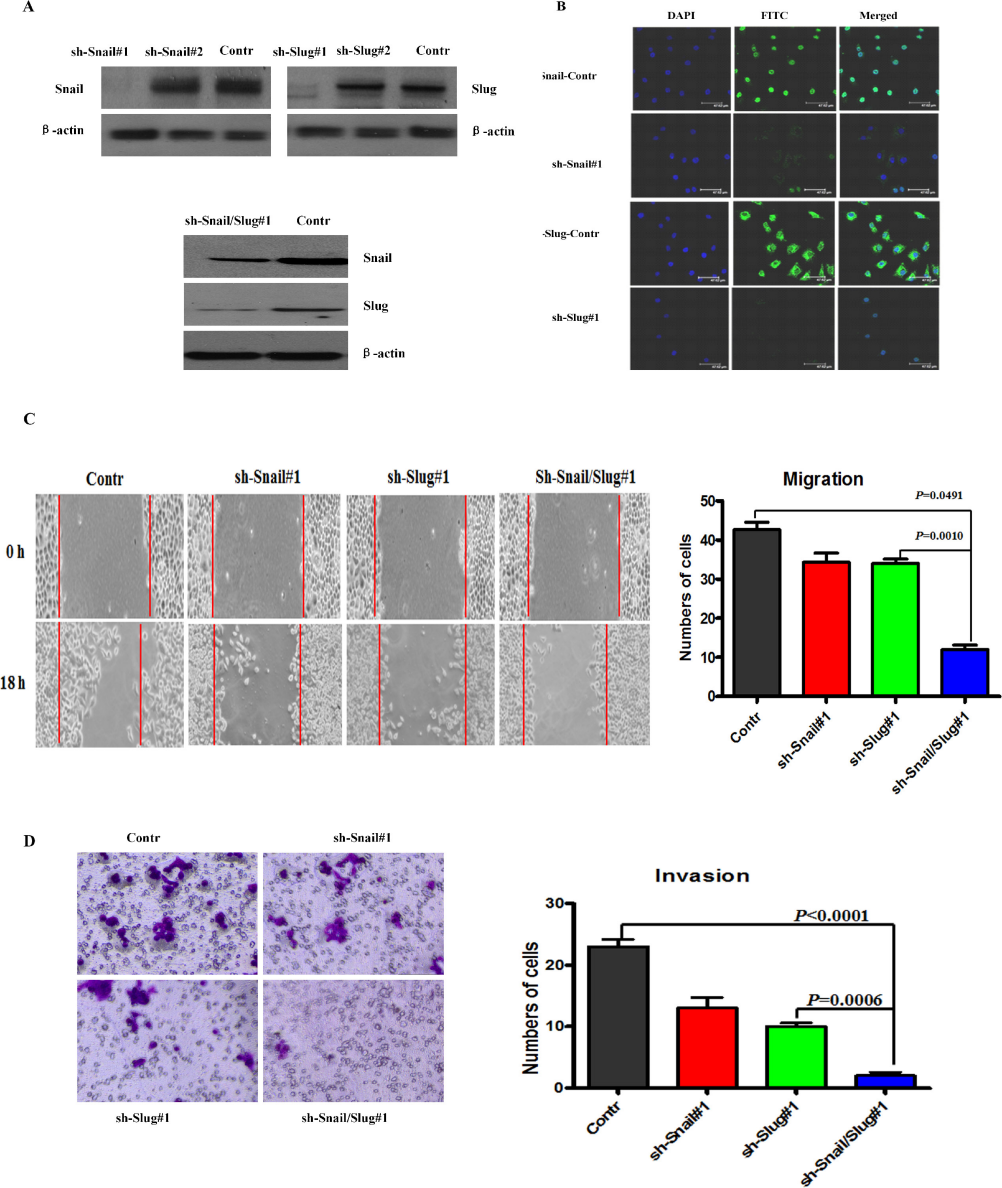
Knockdown of Snail and Slug inhibited the invasion and migration of OTSCC cells **(A)** Immunoblotting assessment of the Snail or Slug protein expression after knockdowning Snail or Slug or both proteins in Cal-27 cells. Representative of three independent experiments was shown. **(B)** Immunofluorescence staining for Snail (sh-Snail#1 and sh-Snail-control) or Slug (sh-Slug#1 and sh-Slug-control). Scale bar, 100 mm. **(C** and **D)** Migration (C) and invasion (D) assays in stable Cal-27 cells transfecting sh-Snail#1 or sh-Slug#1 or sh-Snail/Slug#1. The mean was derived from cell counts of 5 fields, and each experiment was repeated 3 times. Representative images of migrated and invaded cells were shown.

To investigate the roles of Snail and Slug in OTSCC migration and invasion and EMT, we applied short hairpin RNAs (shRNA) to knockdown Snail or Slug or both proteins expression in OTSCC cell line Cal-27, as confirmed by immunoblotting and immunofluorescence staining (Figure 2A, 2B) and real-time PCR (Supplementary Figure S1A). The results showed that knockdown of both Snail and Slug in Cal-27 cells impaired cell migration and invasion ability at 85%–90% (Figure 2C, 2D), compared with control, while knockdown of Snail or Slug decreased cell migration and invasion at 65%–70% (Figure 2C, 2D). And as Snail and Slug are important EMT-inducing transcription factors, we then examined both epithelial and mesenchymal markers by immunoblotting. As can be seen, the silenced cells exhibited a significant upregulation of β-catenin, E-cadherin and Occludin; meanwhile the mesenchymal markers Fibronectin, Vimentin, and N-cadherin were dramatically down-regulated, and more prominently changed in both silenced cells (Supplementary Figure S1B). Real-time PCR analyses also revealed the mRNA expression up-regulation of E-cadherin, Twist2, ZEB1, ZEB2, Prrx1, HOXB7, and downregulation of N-cadherin mRNAs, and more prominently in both silenced cells (Supplementary Figure S1C). Similar results were observed in Tca8113 cells, a prototypic cell model for OTSCC study. These data showed that both Snail and Slug silenced cells have more capacity to inhibit the migratory and invasive behaviors and EMT in OTSCC cells.

**Figure 3:**
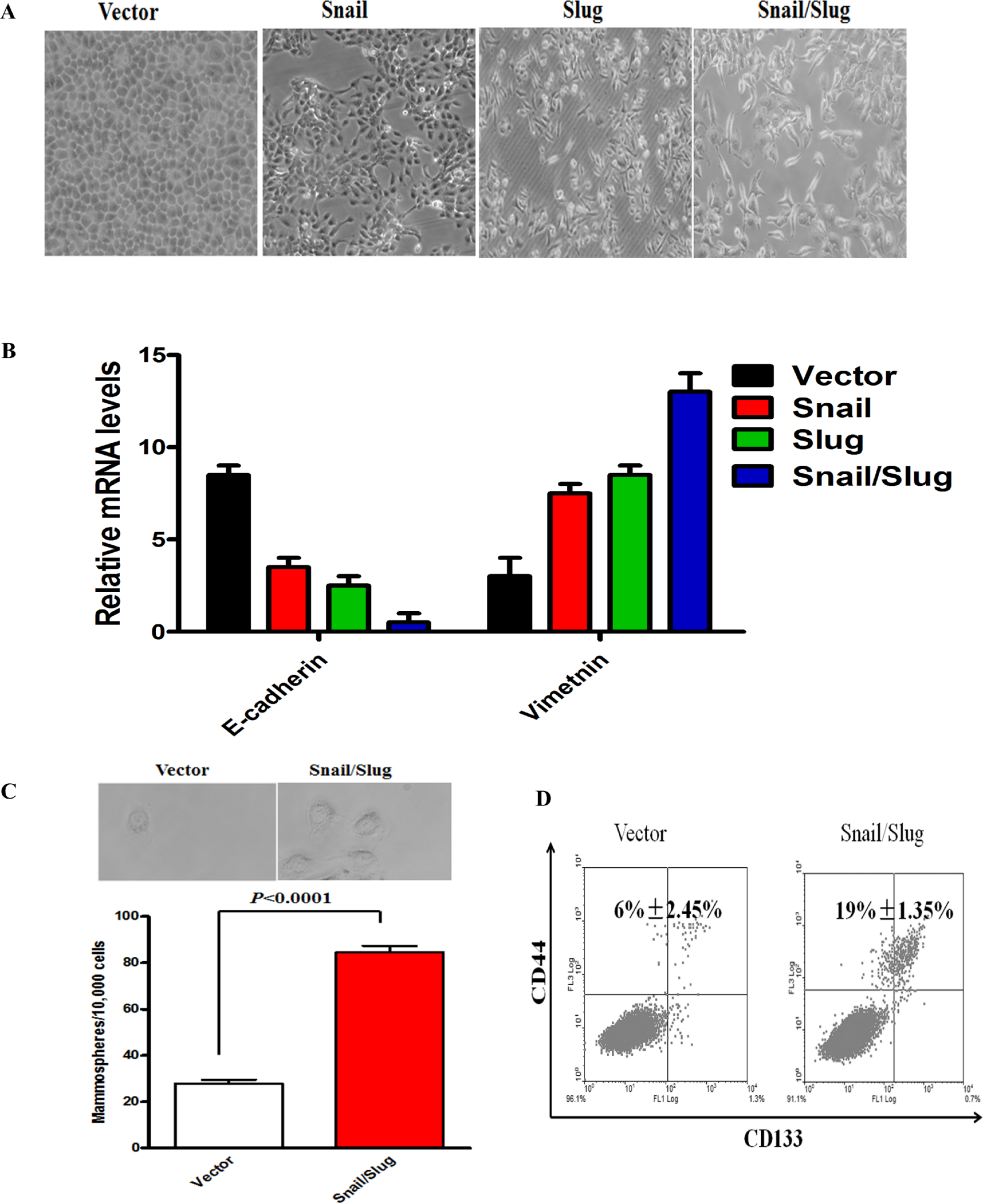
Snail/Slug-induced EMT generated stem cell-like cells **(A)** Morphologic change of Cal-27 cells expressing empty vector, Snail, Slug, or Snail/Slug. Scale bar, 100 mm. **(B)** The results showed that the Snail/Slug-expressing cells exhibited a significant down-regulation of E-cadherin and up-regulation of Vimentin, compared with vector group and Snail group (*p* < 0.05). Snail/Slug-expressing cells had a significant higher level of Vimentin than Slug group (*p* < 0.001), however, there was no difference of E-cadherin expression between Snail/Slug group and Slug group (*p* > 0.05). Error bars represent the mean ± SD of triplicate experiments. **(C)** Phase contrast images and quantification of mammospheres formation. Scale bar, 100 mm. Error bars represent the mean ± SD of triplicate experiments. **(D)** FACS analysis of cell-surface markers CD133 and CD44 in cells expressing Snail/Slug or empty vector. Percentages of mean CD133+/CD44+ subpopulation ± SD based on triplicate experiments are indicated.

To further confirm that the effect of Snail or Slug silencing on the decrease of migration and invasion of Cal-27 cells is specific, we stably overexpressed Snail, Slug, or both proteins in Cal-27 cells (Supplementary Figure S2A). Overexpression of Snail or Slug restored migration and invasion, and cells that co-expressed both proteins demonstrated the highest ability for migration and invasion (Supplementary Figure S2B). The mRNA and protein levels of epithelial markers reduced and the mesenchymal markers increased in Snail or Slug-restored cells, compared with control cells, and co-expressed both proteins gained the highest change of these markers (Supplementary Figure S2C). Importantly, we observed that co-expressed both protein cells displayed an elongated fibroblast-like morphology with scattered distribution in culture, whereas control cells retained their cobblestone-like morphology with tight cell–cell adhesion (Figure 3A). The same results were obtained in Tca8113 cells. Then, we applied RT-PCR to examine the mRNA level of E-cadherin and Vimentin in Snail/Slug groups. The results showed that the Snail/Slug-expressing cells exhibited a significant down-regulation of E-cadherin and up-regulation of Vimentin, compared with vector group and Snail group (*p* < 0.05), Snail/Slug-expressing cells had a significant higher level of Vimentin than Slug group (*p* < 0.001) and there was no difference of E-cadherin expression between Snail/Slug group and Slug group (*p* > 0.05, Figure 3B). These data indicated that both co-overexpression of Snail and Slug contributed to the migratory and invasive behaviors and EMT in OTSCC cells.

Mammary epithelial cells undergoing an EMT program have been linked to stem cell phenotypes such as an increased CD133 high/CD44 high population and mammosphere formation ability [20]. To determine whether co-overexpression of Snail and Slug has the effect to lead to the stem cell phenotypes upon induction of EMT, we carried out FACS to identify CD133 high/CD44 high populations. The CD133 high/CD44 high stem cell population in Cal-27 co-expressed both Snail and Slug proteins were 19% ± 1.35%, and the vector-infected cells was 6% ± 2.45%. The co-overexpression of Snail and Slug Cal-27 cells exhibited a significant increase in the CD133 high/CD44 high stem cell population compared with vector-infected cells (Figure 3D, *p* < 0.05). Meanwhile, as evidenced in Figure 3C, the co-overexpression of Snail and Slug Cal-27 cells increased both in size and in number of mammospheres in comparison with the vector-infected cells. We thus concluded that the co-overexpression of Snail and Slug-induced EMT generates mesenchymal cells with stem cell-like phenotypes, a feature recently defined for EMT inducers.

We next carried out a luciferase reporter assay to investigate whether Snail and Slug could transcriptionally regulate. We transiently transfected cells with luciferase reporter constructs containing proximal promoter of Snail or Slug or E-cadherin. The result showed that the relative luciferase activity of E-cadherin was reduced in cells transiently co-transfected with E-cadherin promoter reporter together with Slug or Snail expression construct, compared with empty vector, indicating that Snail or Slug may directly regulate E-cadherin transcription (Supplementary Figure S3A, S3B). However, the relative luciferase activity of Snail was not changed in cells transiently cotransfected with Snail promoter reporter together with Slug expression construct or with empty vector (Supplementary Figure S3C), indicating that Snail expression could not activate the reporter driven by the Slug promoter. Likewise, Slug expression did not influence the activity of the Snail promoter. These results confirmed the independent expression and regulation of Snail and Slug.

### miR-101 was negatively regulated by both Snail and Slug in OTSCC cells

**Figure 4:**
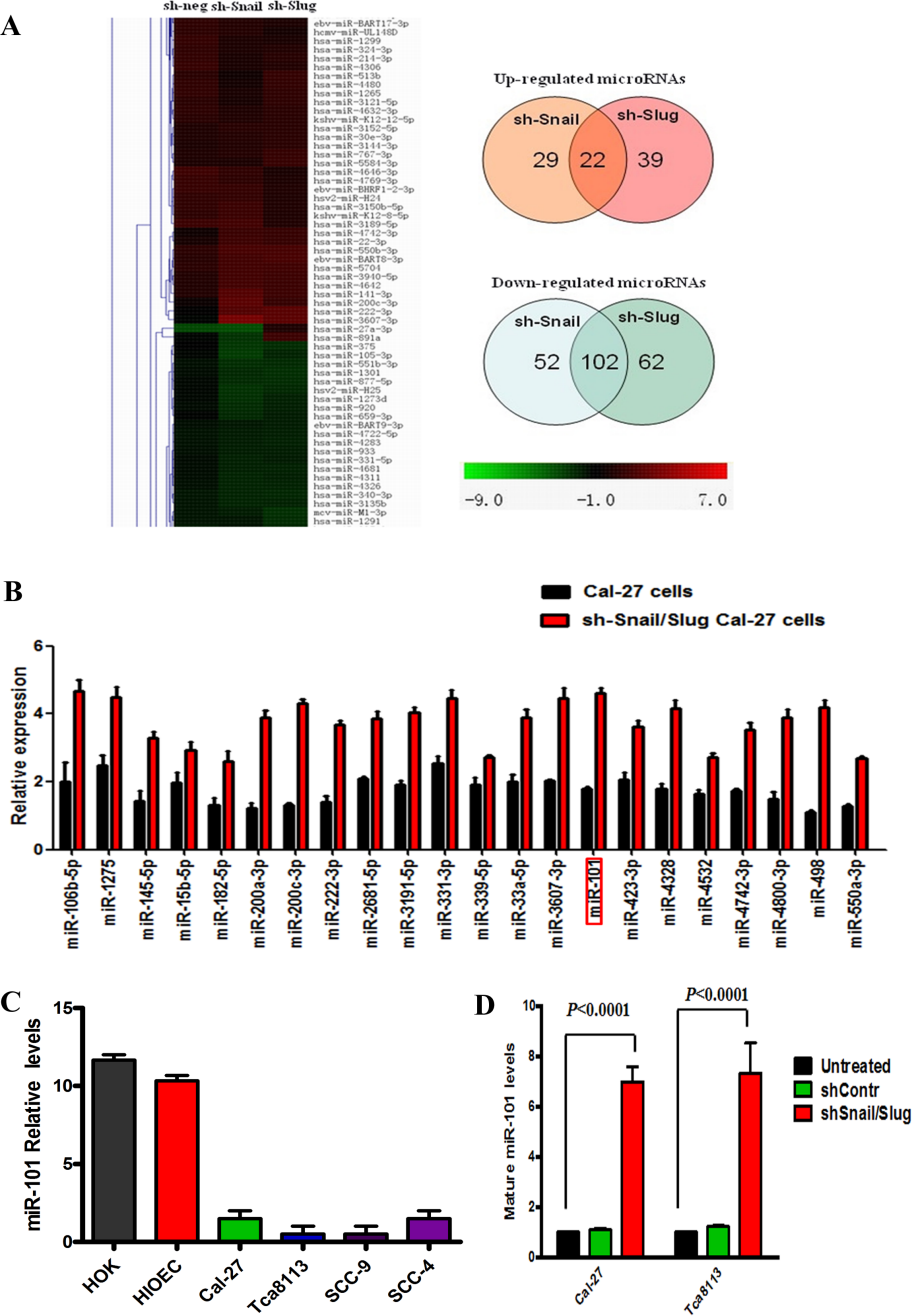
miR-101 was negatively regulated by both Snail and Slug in human OTSCC cells **(A)** miRNA profiling of Cal-27 cells in which Snail or Slug was knocked down. Shades of red represent increased gene expression while shades of green represent decreased expression. 22 miRNAs were jointly up-regulated by both Snail and Slug knockdown. **(B)** Twenty-two miRNAs, including miR-101, were confirmed up-regulated in the sh-Snail/Slug Cal-27 cells by real-time PCR. Error bars represent the mean ± SD of triplicate experiments. **(C)** Mature miR-101 levels assessed by real-time PCR analysis in normal mucous cell lines (HOK and HOEC), and various OTSCC cell lines (Cal-27, Tca8113, SCC-9 and SCC-4). Error bars represent the mean ± SD of triplicate experiments. **(D)** Mature miR-101 expression levels assessed by real-time PCR analysis in Cal-27 and Tca8113 cells that were treated for 72 hr with sh-negative or sh-Snail or sh-Slug. Error bars represent the mean ± SD of triplicate experiments.

To investigate the functional relationship between Snail and Slug in silencing tumor-suppressive miRNAs in OTSCC, we compared the miRNA expression profile of OTSCC cells in which Snail or Slug had been knocked down with that of control cells. The results showed that 29 miRNAs were suppressed by Snail and 39 were suppressed by Slug. Notably, twenty-two miRNAs were up-regulated in both knockdown cells (Figure 4A). Real-time PCR verification was performed to verify the miRNA array results (Figure 4B). Among these, miR-101 attracted our attention for the following reasons. Down-regulated expression of miR-101 has been found in a variety of cancers, and associated with the invasion and progression of malignancies. Its gene therapy is of great potentiality to be a new modality for treatment of cancers [21]. Nevertheless, the expression of miR-101 in OTSCC tissues and cells, as well as the mechanisms underlying miR-101 silencing in OTSCC remain largely unknown [22].

Next, we performed quantitative real-time PCR (qRT-PCR) assays and found that miR-101 level was widely repressed in OTSCC cell lines as compared with those in human oral keratinocytes cell lines and human immortalized oral epithelial cell (Figure 4C). To validate the results obtained from miRNA microarray experiments, we knocked down Snail or Slug in Cal-27 and Tca8113 cells. qRT-PCR assays confirmed that, upon knockdown of both gene, the expression level of miR-101 was significantly increased in both cell lines (Figure 4D). To investigate the mechanism of Snail/Slug repression of miR-101, we applied a luciferase reporter assay to examine whether miR-101 could transcriptionally regulate the Snail/Slug expression. We found that the relative luciferase activity of Snail was decreased proportionally to the increasing amount of miR-101 in cells (Supplementary Figure S4A). However, the Slug promoter activity was not changed (Supplementary Figure S4B). These findings suggest that miR-101 may directly regulate Snail transcription and indirectly regulate Slug transcription.

### miR-101 suppressed the invasion and migration, and EMT of OTSCC cells by targeting EZH2

**Figure 5:**
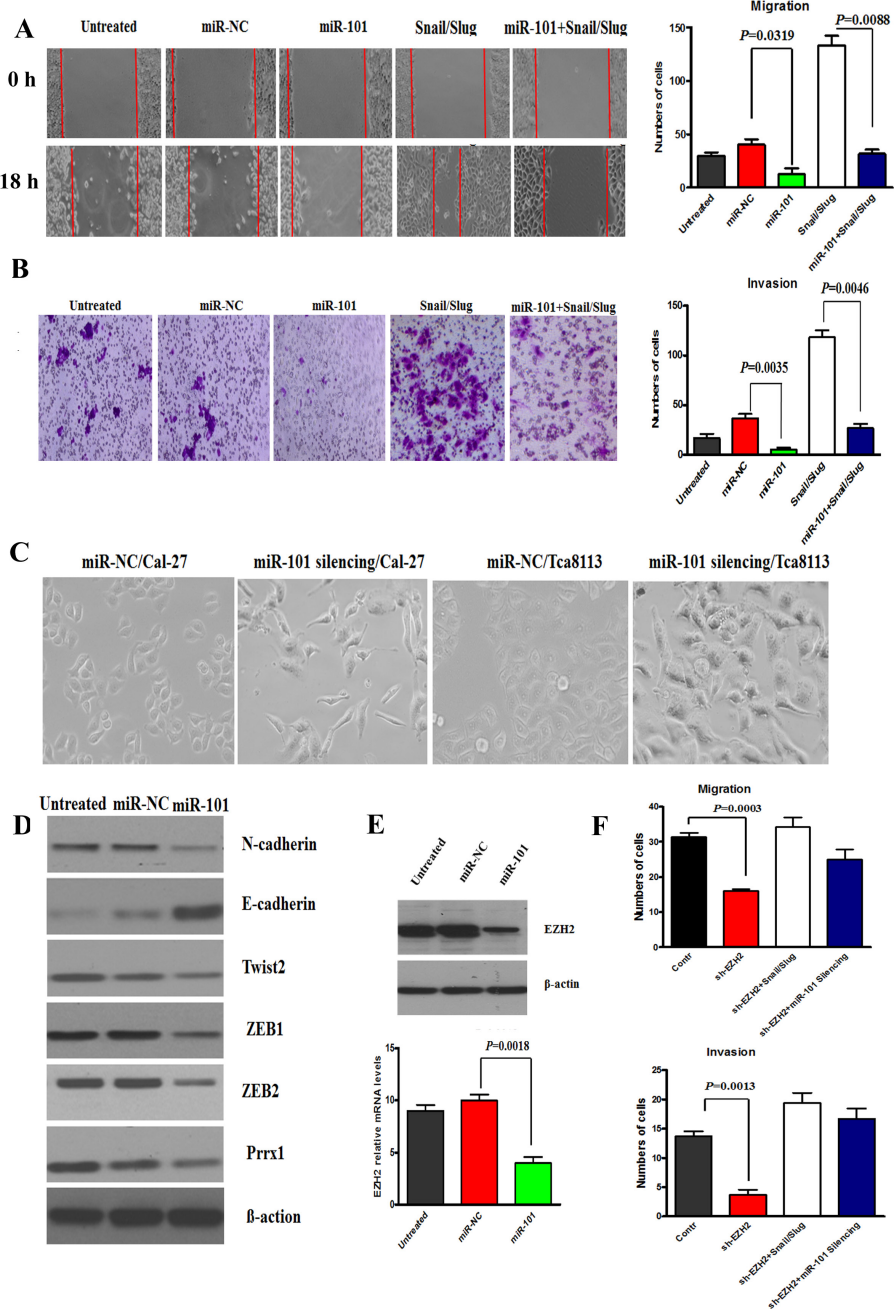
miR-101 restoration reversed the invasion, migration and EMT of Cal-27 cells **(A** and **B)** Migration (A) and Invasion (B) assay of Cal-27 cells untreated or treated with 100 nM miR-NC or miR-101 for 48 hr or miR-101 and Snail/Slug or Snail/Slug. The mean was derived from cell counts of 5 fields, and each experiment was repeated 3 times. Representative images of migrated and invaded cells were shown. **(C)** Cal-27 and Tca8113 cells with miR-101 silencing showed an obvious shift in morphology, from cobblestone-like cells to more spindleshaped cells. Scale bar, 50 mm. **(D)** Western Blot analysis revealed a significant down-expression of N-cadherin, Twist2, ZEB1, ZEB2, Prrx1 and up-expression of E-cadherin in miR-101 overexpression cells. Representative of three independent experiments was shown. **(E)** The protein and mRNA levels of EZH2 expression in Cal-27 cells overexpressing miR-101 were reduced. Error bars represent the mean ± SD of triplicate experiments. **(F)** Migration and invasion assays of Cal-27 cells with EZH2 knockdown, EZH2 knockdown with Snail/Slug overexpression, and EZH2 knockdown with miR-101 silencing. The mean was derived from cell counts of 5 fields, and each experiment was repeated 3 times.

Next, we aimed at investigating the effects of miR-101 expression level on the malignant phenotype of OTSCC cells. The results showed that miR-101 restoration significantly reduced the migration and invasion ability of Cal-27 cells (Figure 5A, 5B) and blocking the endogenous miR-101 expression significantly enhanced the migration and invasion ability, supporting the tumor suppressor role of miR-101 in OTSCC cells. Further, we stably overexpressed miR-101 in Snail/Slug overexpression cells and the migration and invasion of cells were examined. The data showed that the overexpression of miR-101 significantly inhibited the migration and invasion in Snail/Slug overexpression cells (Figure 5A, 5B). This indicated that miR-101 is a downstream target of Snail/Slug-induced migration and invasion in OTSCC. Comparing with control cells, Cal-27 and Tca8113 cells with miR-101 silencing showed an obvious shift in morphology, from cobblestone-like cells to more spindleshaped cells (Figure 5C). To further probe the possible interactions between miR-101 and EMT-inducing transcription factors, we examined the expression of known EMT inducers. We showed that the mRNA levels of N-cadherin, Twist2, ZEB1, ZEB2, Prrx1 were reduced in response to miR-101 overexpression, whereas E-cadherin mRNA level was increased (Supplementary Figure S5). Western Blot analysis revealed a significant increase of E-cadherin expression and reduced Vimentin expression in miR-100 overexpression cells (Figure 5D). These results indicate that miR-101 is required for EMT maintenance in mesenchymal phenotype cells.

We also investigated the effects of miR-101 level on EZH2 gene expression, a well-known tumor-promoting gene, recently described as an important miR-101 target [22]. Both at transcriptional and post-transcriptional levels, we observed a reduced EZH2 expression in Cal-27 cells overexpressing miR-101 (Figure 5E) and an increased EZH2 expression in Cal-27 cells characterized by miR-101 inhibition, confirming that EZH2 may be a direct target of miR-101. Next, we asked whether EZH2 was responsible for the aggressive phenotype of OTSCC cells. We found that EZH2 knockdown significantly inhibited the migration and invasion of Cal-27 cells (Figure 5F), and the mRNA levels of N-cadherin, Twist2, ZEB1, ZEB2, Prrx1 were reduced in response and E-cadherin mRNA level was increased. These data suggested that EZH2 may be a target of miR-101 in suppressing the migration and invasion, and EMT of OTSCC cells. Further, we stably knocked down EZH2 in Snail/Slug overexpression cells and the migration and invasion of cells were examined. The data showed that there was no difference of the migration and invasion between control and sh-EZH2 cells with Snail/Slug overexpression (*p* > 0.05), indicating that the knockdown of EZH2 significantly inhibited the invasion and migration in Snail/Slug overexpression cells (Figure 5F). And we stably knocked down EZH2 in miR-101 silencing cells. The data demonstrated that the knockdown of EZH2 also significantly inhibited the invasion and migration in miR-101 silencing cells (Figure 5F). These data indicated that EZH2 may mediate Snail and Slug/miR-101-induced migration and invasion in OTSCC cells.

### Co-overexpression of Snail and Slug was associated with lower miR-101 and higher EZH2 levels of OTSCC patients

**Figure 6:**
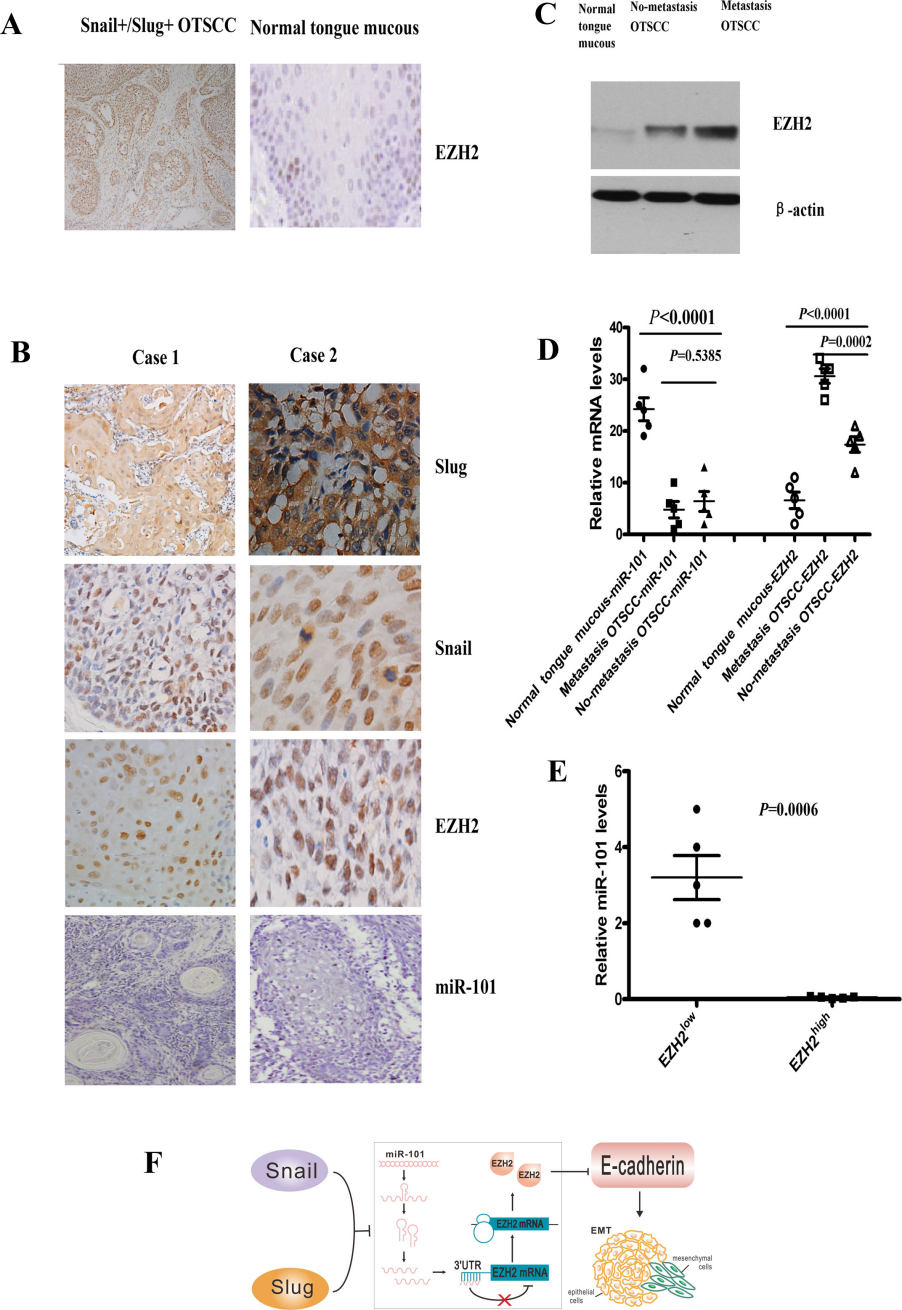
Co-overexpression of Snail and Slug was associated with lower miR-101 and high EZH2 levels of OTSCC patients **(A)** Representative images of immunohistochemical staining for EZH2 in serial sections of OTSCC patients and normal tongue mucous. Scale bar, 100 mm. **(B)** *In situ* hybridization for miR-101 and immunohistochemical staining for Snail, Slug and EZH2 in case 1(T1N0M0), case 2 (T1N1M0) of OTSCC patients. Scale bar, 50 mm. **(C)** EZH2 protein expression in metastasis OTSCC, no-metastasis OTSCC and normal tongue mucous by Western blot. Representative of three independent experiments was shown. **(D)** Relative mRNA levels of miR-101 and EZH2 in metastasis OTSCC, no-metastasis OTSCC and normal tongue mucous by real time RT-PCR. Error bars represent the mean ± SD of triplicate experiments. **(E)** Relative miR-101 levels assessed by real time RT-PCR in OTSCC according to the expression levels of EZH2. EZH2^low^ means the expression of EZH2 in normal tongue mucous, and EZH2^high^ means the expression of EZH2 in metastasis OTSCC. **(F)** Schematic diagram of Snail and Slug collaboration of EMT and metastasis through miR-101-mediated EZH2. The Snail and Slug/miR-101/EZH2 pathway would be regarded as a novel regulatory axis of EMT-mediated-microRNA signaling.

To further investigate the impact of co-overexpression of Snail and Slug on EZH2 expression of OTSCC patients, we performed IHC analysis to examine EZH2 expression in consecutive tissue slides of 30 Snail^+^/Slug^+^ OTSCC samples, and 10 normal human tongue mucous tissues. The overexpression of EZH2 was 29/30 (96.67%) of Snail^+^/Slug^+^ OTSCC tumors, whereas no obvious expression of EZH2 was observed in any of the 10 normal tongue mucous samples (Figure 6A). EZH2 levels in Snail^+^/Slug^+^ OTSCC were significantly higher than the normal mucous (*p* < 0.0001).

To confirm the association of co-overexpression of Snail and Slug with the level of EZH2 in OTSCC, we performed IHC staining for another independent OTSCC patient cohorts. The cohorts (Zhoushan), consisted of patients of a current prospective study with a median follow-up of 12.5 months (range, 3–50 months; *n* = 40). IHC staining of Snail, Slug and EZH2 in metastasis and no-metastasis OTSCC patients revealed similar findings (Supplementary figure S6A, Table S4). Co-overexpression of Snail and Slug reliably predicted the clinical outcome (Supplementary Figure S6B; *p* < 0.05), and confirmed the positive correlation of co-overexpression of Snail and Slug with EZH2 (*p* < 0.001).

To quantify the correlation between miR-101 and EZH2 in OTSCC samples, we examined the protein and mRNA expression levels of miR-101 and EZH2 in fresh samples from five metastatic OTSCC, five no-metastatic OTSCC, and five normal tongue mucous tissues (Figure 6B, 6C, 6D). The protein expression of miR-101 was totally lost in OTSCC by *in situ* hybridization (Figure 6B), and all normal tongue mucous tissues assayed exhibited higher mRNA levels of miR-101, metastasis OTSCC and no-metastatic OTSCC showed lower levels of miR-101 (Figure 6D). As expected, metastatic OTSCC expressed significantly higher levels of EZH2 as compared to no-metastatic OTSCC or normal tongue mucous tissues (*p* = 0.0002, *p* < 0.0001, respectively, Figure 6C, 6D). Consistent with a functional connection between miR-101 and EZH2, miR-101 expression was significantly decreased in metastatic OTSCC relative to no-metastatic OTSCC or normal tongue mucous tissues (*p* = 0.0006, Figure 6E). Taken together, these data strongly suggest that co-overexpression of Snail and Slug was closely associated with lower miR-101 and higher EZH2 levels of OTSCC patients (Figure 6F).

## DISCUSSION

EMT has been considered the critical mechanism involved in cancer metastasis and EMT transcription factors could collaborate with each other [23], however, the interplay mechanism of EMT transcription factors in human cancers are largely unknown. Here, we identified a novel mechanism in which co-overexpression of Snail and Slug can recruit EZH2 by silencing of tumor-suppressive miR-101, thus establishing a functional link between aberrant expression of Snail and Slug and global down-regulation of miRNAs in OTSCC. These results not only pinpoint the role of co-expression of Snail and Slug in regulating miRNAs in OTSCC, but also demonstrate that EMT transcription factors can mediate miRNAs and further induce EMT and metastasis.

As strong E-cadherin repressors and major EMT inducers, Snail and Slug play a critical role in the invasion and metastasis of plenty of human cancers [5–8]. However, the interplay between Snail and Slug in the promotion of EMT in OTSCC has not been reported. In the present study, we provide evidence that Snail and Slug are regulated independently and act collaboratively to promote EMT: 1) co-overexpression of Snail and Slug indicates the worst prognosis for OSTCC patients; 2) OSTCC cell lines harboring both Snail and Slug showed a higher migratory and invasion ability; 3) co-overexpression of Snail and Slug can generate CD133 high/CD44 high stem cell-like cells; 4) Snail and Slug did not influence the expression/promoter activity of each other, suggesting that Snail may not be able to bind directly to the Slug promoter; rather, it may indirectly activate the Slug promoter through as yet unidentified intermediate factors. This was the first report to demonstrate the independent regulation of Snail and Slug and their additive effects on EMT promotion of OTSCC. The results were in line with the reports that many EMT-inducing transcription factors are often activated simultaneously, such as expression of Twist1, Snail, Snail2 and ZEB2 in neural crest cells [24, 25]. Castro Alves et al. [26] found that Slug might act synergistically with Snail and SIP1 in the down-regulation of E-cadherin in 59 gastric carcinomas tissues, and Casas et al. [27] showed that Twist1 and Snail2 acted together to promote EMT and breast tumor metastasis. These data implicated the interactions among these EMT inducers, however, the mechanism that the transcription factors, especially Snail and Slug, coordinated the EMT program to promote tumor metastasis remained largely unknown.

Although some miRNAs are up-regulated during tumorigenesis [28], a global reduction of abundant miRNAs appears a general feature of human cancers, and plays a causal role in the process of malignant transformation as oncogenes or tumor suppressors [29–31]. In this study, microRNA microarray expression profiling showed that the expression and function of Snail and Slug complex were under rigorous surveillance of 22 tumor-suppressive miRNAs. Further, we found that the Snail and Slug complex target, miR-101, regulated the migration and invasion of OTSCC cells. As an important tumor-suppressive miRNA, plenty of data demonstrated that miR-101 expression was reduced in several human cancers [32–36] and could inhibit the expression of tumor promoting genes [37–39]. During the process of OTSCC, we showed that the Snail and Slug complex was activated and miR-101 was silenced, resulting the migration and invasion, EMT of OTSCC cells. Thus, the process of OTSCC would be accelerated upon miR-101 loss.

Then we found that miR-101 silencing could lead to EZH2 overexpression. Although miR-101 based regulation over EZH2 has been addressed independently in human cancers [37, 40–41], the interaction between Snail/Slug, miR-101 and EZH2 remains to be systematically investigated. In the present study we explored that Snail and Slug up-regulated EZH2 by down-regulating miR-101 in OTSCC. EZH2 is the sole histone methyltransferase that catalyzes trimethylation of histone H3 at lysine 27 (H3K27me3), thereby mediating epigenetic gene silencing [42]. Although mounting evidence suggests that EZH2 was aberrantly elevated in a subset of aggressive solid tumors including breast cancer [43], melanoma [44], bladder cancer [45], gastric cancer [46] and other cancers [47], recent studies in myeloid malignancies and lymphomas show that EZH2 has a tumor suppressor role [48], suggesting that the role of EZH2 in cancer development varies with different types of cancer [49]. Our studies show that EZH2 has an oncogenic role in HNSCC, and co-overexpression of Snail and Slug is closely associated with lower miR-101 and high EZH2 levels of OTSCC patients. However, there is no difference between early and advanced tumor stage relative to EZH2 expression in HNSCC reported by Banerjee group [22]. The discrepancy might be due to different histological characteristics of the chosen tumors. More work should be done.

HNSCC accounts for about 80% of all oral malignant tumors. The 5-year survival rate for patients with HNSCC was still poor, approximately 56%, predominantly because of widespread lymphatic and distant metastasis [50, 51]. Recent studies have linked EMT with OTSCC. Targeting the EMT-like phenotypes would seem to represent the potential strategy for the development of novel anticancer therapeutics. Our results in this study are in favor of a strong inverse correlation between Snail/Slug and miR-101, and the loss of miR-101 and concomitant elevation of EZH2 is most pronounced in metastatic cancer. Hence, we postulate that approaches to silence Snail/Slug and re-introduce miR-101 into OTSCC may have therapeutic benefit by reverting the epigenetic program of tumor cells to a more normal state.

## MATERIALS AND METHODS

### Patients and specimens

Patients with OTSCC who were diagnosed, treated, and followed at the Department of Oral and Maxillofacial Surgery, West China Hospital of Stomatology (Sichuan University) between 2002 and 2005 were recruited for the present study after giving informed consent. Inclusion criteria were primary resection, no prior treatment, complete medical records and the availability of all the histopathologic slides of the resection specimens. Finally, 89 patients, 49 male and 40 female, were recruited in this study, demographic and other variables were retrieved from the database provided by the tumor registry (Table 1). The protocol of the study was approved by the Institutional Ethics Committee of the West China Medical Center, Sichuan University, China.

### Immunohistochemical staining

Antigen retrieval and staining with polyclonal antibodies were used to identify the markers in cancer cells. All the sections were dewaxed and rehydrated. Endogenous peroxidase activity was blocked by incubation with 3% hydrogen peroxide for 20 min. Antigen retrieval was accomplished by 0.01 M citrate buffer solution (pH = 6.0) in a microwave oven 700 W for 15 min. The sections were washed in phosphate-buffered saline (PBS) for 5 min twice, and then incubated with 5% normal goat serum for 20 min. The slides were then exposed in a humid chamber for 1 hour at 37°C and overnight at 4°C to the primary antibodies against Snail and Slug. Sections were then incubated with biotinylated anti-goat IgG and streptavidin-biotin peroxidase. Diaminobenzidine tetrahydrochloride (DAB) was used to detect antigen-antibody binding, and the slides were counterstained with haematoxylin. Nuclear expression of Snail and cytoplasm staining of Slug were graded as positive expression.

### Cell lines and cell culture

Human squamous cell carcinoma of tongue cancer cell lines, Cal-27, Tca8113, SCC-9 and SCC-4, were provided by State Key Laboratory of Oral Diseases, Sichuan University. Cells were cultured in RPMI 1640 medium (Gibco) supplemented with 10% heat-inactivated FCS (Hyclone), 2 mmol/L L-glutamine, 25 mmol/L HEPES, and 100 units/mL penicillin and streptomycin in a humidified 5% CO_2_ atmosphere.

### Cloning, lentivirus preparation, and plasmids

The lenti-X shRNA expression system (Clontech) was used for the construction of the lentivirus expression construct according to the manufacturer's instruction. Short pairs of sense and antisense DNA oligo encoding a sense-loop-antisense sequence to Snail and Slug genes were synthesized for the validated corresponding siRNAs, and sequences are listed as following: shRNA-Snail: 5′-aggccttcaactgcaaata-3′; shRNA- Slug: 5′-ggaccacagtggctcagaa-3′; and shRNAnegative-: 5′-actaccgttgttataggtg-3′. The cDNA oligos were annealed and cloned to the BamH I/EcoR I-digested pLVX-shRNA1 vectors (Clontech). The recombinant vectors were purified and cotrans fected with Lenti-X HT packaging Mix (Clontech) into HEK 293T packaging cells using Lenti-X™ HTX Packaging System (Clontech). The virus-containing cell culture supernatants were collected 48 hours after transfection, passed through a 0.45-μm filter, and stored at –80°C. The virus titration was determined using puromycin selection following the manufacturer's protocol. After 24 hours, the OTSCC cells were infected with recombinant lentivirus vectors at a multiplicity of infection of 5.

The full-length Snail and Slug cDNA was cloned into the pcDNA3 plasmid vector and transfected into cells by LipofectAMINE reagent (Invitrogen) according to the manufacturer's instructions. Stable transfected cells were selected in 400 μg/mL Geneticin and further subcloned.

### Transfection of miRNA mimics and inhibitors

A total of 2 × 10^5^ cells were plated in triplicate overnight in antibiotic-free complete medium in 6-well plates. The cells were then transiently transfected with 200 μL containing 100 nM of a mature miRNA mimic or inhibitor (Applied Biosystems) using Lipofectamine 2000 (Invitrogen) according to the manufacturer's protocol. 72 h after transfection, the total RNA and protein were collected for further analysis.

### Immunofluorescence assay

Cells were seeded onto coverslips at a density of 10^4^/mL and cultured in a 6-well culture plate for 24 hours. Cells grown on coverslips were washed in cold PBS and fixed in 2% paraformaldehyde-PBS for 20 minutes, permeabilized in 0.5% Triton X-100 in PBS for 10 minutes at 4°C, and blocked in 1% bovine serum albumin for 30 minutes at room temperature. Coverslips were incubated overnight with primary antibodies, followed by incubation with TRITC-conjugated secondary antibodies for 1 h, and then stained with DAPI. Finally, coverslips were observed under a fluorescence microscope (Olympus BX51).

### Western blot

Total proteins were isolated from the cultured monolayer cells with a total protein extraction kit (Keygen), and protein concentrations were detected by a bicinchoninic acid protein assay kit (Pierce). Thirty-microgram proteins from each sample were separated on 8% SDS-PAGE and transferred electrophoretically to polyvinylidene difluoride membranes (Millipore). Membranes were blocked with 2% bovine serum albumin in TBS containing 0.1% Tween20 (TBST) at 37°C for 2 hours and then incubated for 2 hours respectively with primary antibody. Horseradish peroxidase–conjugated antimouse or antirabbit IgG were used as secondary antibody. Bands were scanned using a densitometer (GS-700, Bio-Rad Laboratories), and quantification was done using Quantity One 4.4.0 software.

### Quantitative real-time reverse transcriptase-PCR

Total RNA was isolated with TRIzol reagent (Invitrogen) and treated with RNase-free DNase I (Takara) to avoid genomic DNA contamination. PCR amplification of the cDNA template was done using Thunderbird SYBR qPCR mix (TOYOBO) on ABI PRISM 7300 sequence detection system (Applied Biosystems). Reactions were run in triplicate, and results were averaged. Each value was normalized to GAPDH as the housekeeping gene to control for variations in the amount of input cDNA. The relative expression level of the genes was calculated using the 2^−ΔΔCT method.

### Wound-healing assay

Cells were plated in 6-well plates at 2.0 × 10^5^ cells/well. When cells reached 80% confluence, the individual wells were wounded by scratching with a pipette tip and incubated with medium containing no FBS to 0, 18 h. Cells were photographed under phase-contrast microscopy (×10).

### Transwell invasion assays

*In vitro* cell invasion assays were performed with QCMTM 96-well cell invasion assay kit (Chemicon International, Temecula, CA, USA). 5 × 10^4^ cells were seeded into the top chamber coated with Matrigel (BD Biosciences). Complete medium was added to the bottom wells to stimulate invasion. After cells were incubated for 24–48 h, they were stained with 0.1% Crystal Violet. The cells that had invaded through matrigel and reached to the reverse side were counted under a microscope in five pre-determined fields at a magnification of × 200. Each assay was performed in triplicate.

### Luciferase reporter assay

Cells of 50%–90% confluence in 48-well plates were transfected using Lipofectamine 2000 (Invitrogen). Firefly luciferase reporter gene construct (100 ng) and a Renilla luciferase construct (5 ng; for normalization) were co-transfected per well. Cell extracts were prepared 48 h after transfection, and the luciferase activity was measured using the Dual-Luciferase Reporter Assay System (Promega).

### Mammosphere formation assays

Single cells were plated at 10,000 cells/mL on 6-well ultra-low attachment plates (Corning) in serum-free DMEM/F12 supplemented with 20 ng/mL bFGF, 20 ng/mL EGF, 4 mg/mL insulin, 4 mg/mL heparin, 1 mg/mL hydrocortisone, 0.4% BSA and B27. Fresh medium was supplemented every 3 days. The mammospheres were counted at day 14.

### Flow cytometry

A total of 1 × 10^6^ cells were resuspended in 100 mL PBS containing 2% FBS (FACS buffer), and then incubated on ice for 10 minutes. CD133-APC and CD44- PE (BD Biosciences) were added to cell suspension and incubated on ice for 30 minutes. Cells were washed and resuspended in 500 mL FACS buffer and analyzed using a FACS Calibur Flow Cytometer (Cytomic FC500, Beckman).

### microRNA microarray and data analysis

Total RNA from cells was isolated using TRIzol reagent (Invitrogen, Carlsbad, CA, USA) following the manufacturer's instructions. Two mg of total RNA from each sample were labeled using the miRCURY^TM^ Hy3^TM^/Hy5^TM^ labelling kit and hybridized on the miRCURYTM LNA Array (v.8.0) (Exiqon, Vedbaek, Denmark). Signal intensities were normalized using the global Lowess regression algorithm. For subsequent analysis, we used the log2 of the background-subtracted, normalized median spot intensities of ratios from the two channels (Hy3/Hy5). To find consistently differentially expressed genes, the data were subjected to SAM analysis.

### miRNA expression analysis

Total RNA was isolated from cell lines using TRIzol (Invitrogen). Reverse transcription was performed using miScript Reverse Transcription Kit (Qiagen) and real-time PCR was performed in triplicate on CFX96 Real-Time PCR Detection System (Bio-Rad), using the SYBR Premix Ex Taq (Takara). The 2^-ΔΔCT method was used to determine the relative gene expression, with miRNA levels normalized to U6 small nuclear RNA. Primer of miR-101 is CTACAGTACTGTGATAACTGAA and RNU6B_2 miScript Primer (QIAGEN).

### Statistical analyses

Chi-square was used to analyze the associations between Snail and Slug expression and clinical-pathological characteristics. The Kaplan–Meier method was applied for the overall survival and disease-free survival and the statistical significances between the groups were evaluated using the log-rank test. Univariate and multivariate analyses were performed by means of the Cox proportional hazards model. Variables with *p* value < 0.05 after the univariate analysis were entered into multivariate analysis with a forward stepwise selection. The comparisons of means among groups were analyzed by one-way ANOVA, and the Dunn's Multiple Comparison Test was further used to determine the specific differences between groups. All statistical analyses were done using the SPSS package (version 13.0). A value of *p* < 0.05 was considered statistically significant.

## SUPPLEMENTARY FIGURES AND TABLES


