# Supplementary material for: Snail and Slug collaborate on EMT and tumor metastasis through miR-101-mediated EZH2 axis in oral tongue squamous cell carcinoma
Source: Oncotarget. 2015 Feb 9;6(9):6794–810. doi: 10.18632/oncotarget.3180 (PMC4466650; doi:10.18632/oncotarget.3180)
Supplement: Supplementary file 1 [file oncotarget-06-6794-s001.pdf]

SUPPLEMENTARY FIGURES AND TABLES

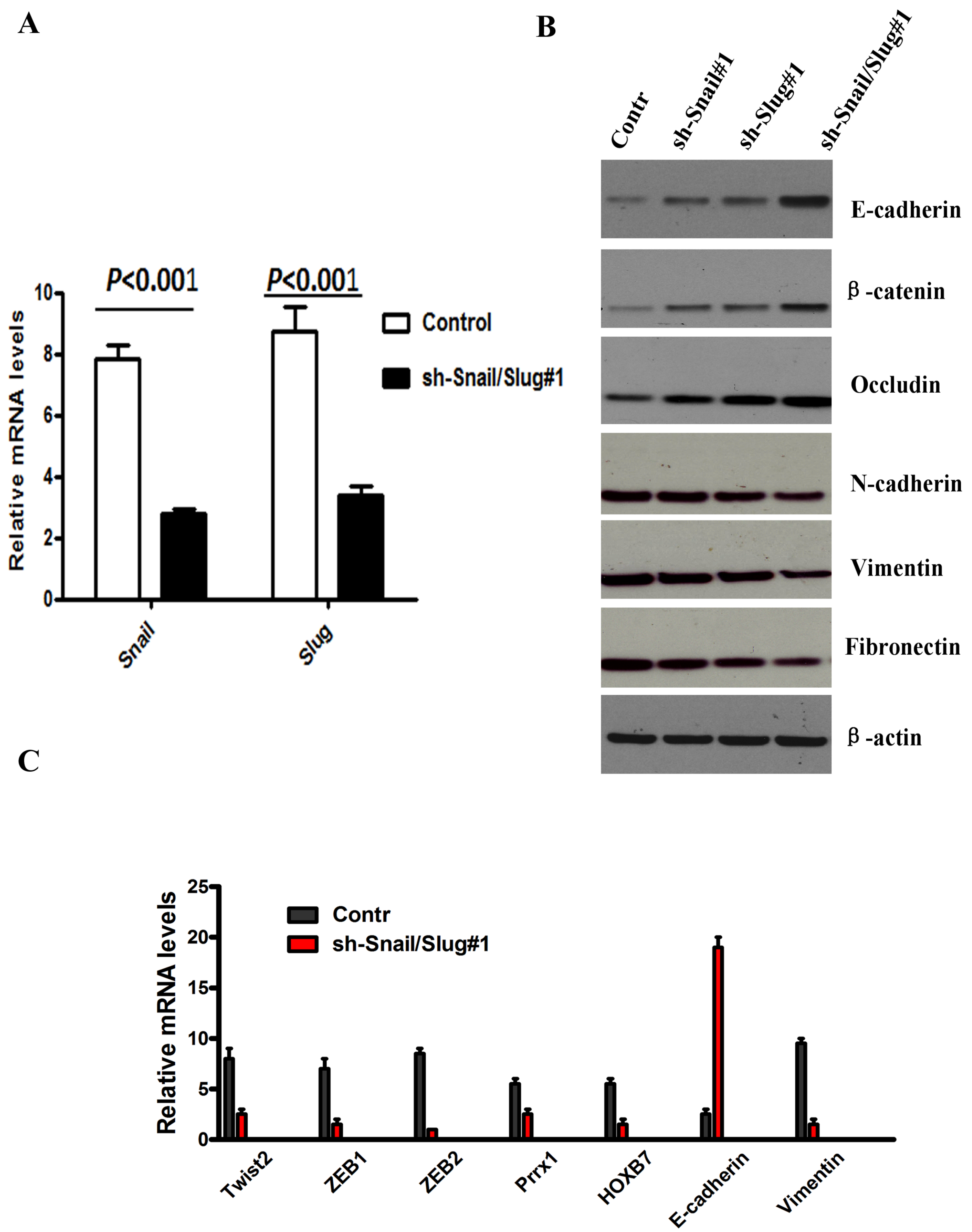

Supplementary Figure S1: Knockdown of Snail and Slug inhibited EMT of OTSCC cells. (A) Short hairpin RNAs (shRNA) to knockdown Snail and Slug in Cal-27 cells was assessed by real-time PCR. Error bars represent the mean ± SD of triplicate experiments. **(B)** The expression of E-cadherin, β-catenin, Occludin, Fibronectin, Vimentin, and N-cadherin mRNAs in Cal-27 cells with knockdowning Snail and Slug were assessed by Western blot. Representative of three independent experiments was shown. **(C)** The mRNA expression levels of known EMT inducers in Cal-27 cells with knockdowning Snail and Slug were assessed by real-time PCR. Error bars represent the mean ± SD of triplicate experiments.

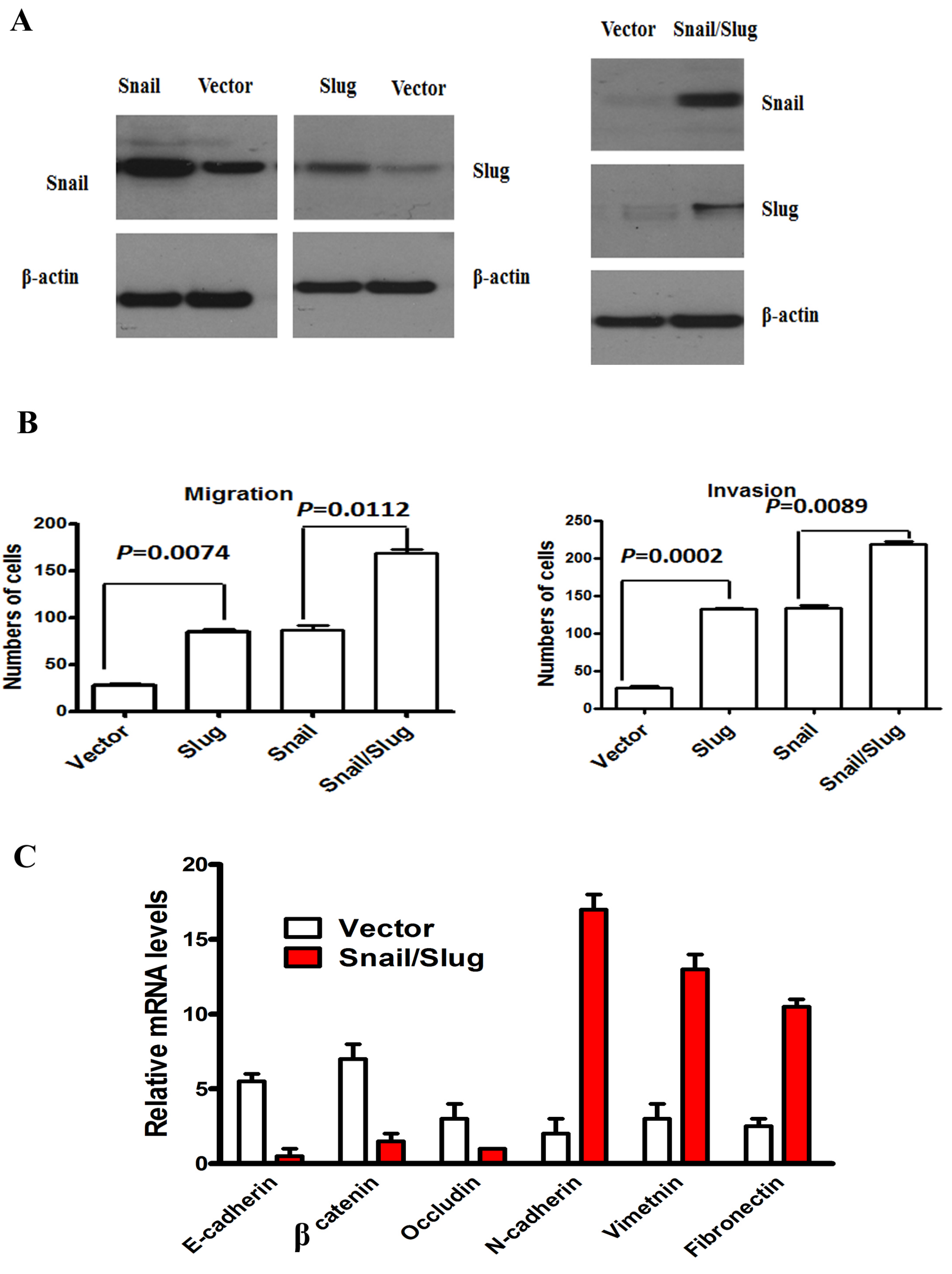

Supplementary Figure S2: Overexpression of Snail or Slug restored migration, invasion and EMT, and cells that co-expressed both proteins demonstrated the highest ability for migration and invasion. **(A)** Overexpressed Snail, Slug, or both proteins in Cal-27 cells were assayed by Western blot. Representative of three independent experiments was shown. **(B)** Migration (B Right) and invasion (B Left) assays in stable Cal-27 cells transfecting Snail or Slug or Snail/Slug. The mean was derived from cell counts of 5 fields, and each experiment was repeated 3 times. **(C)** The mRNA levels of E-cadherin, β-catenin and Occludin reduced and N-cadherin, Vimentin and Fibronectin increased in Snail and Slug-restored cells, compared with control cells. Error bars represent the mean ± SD of triplicate experiments.

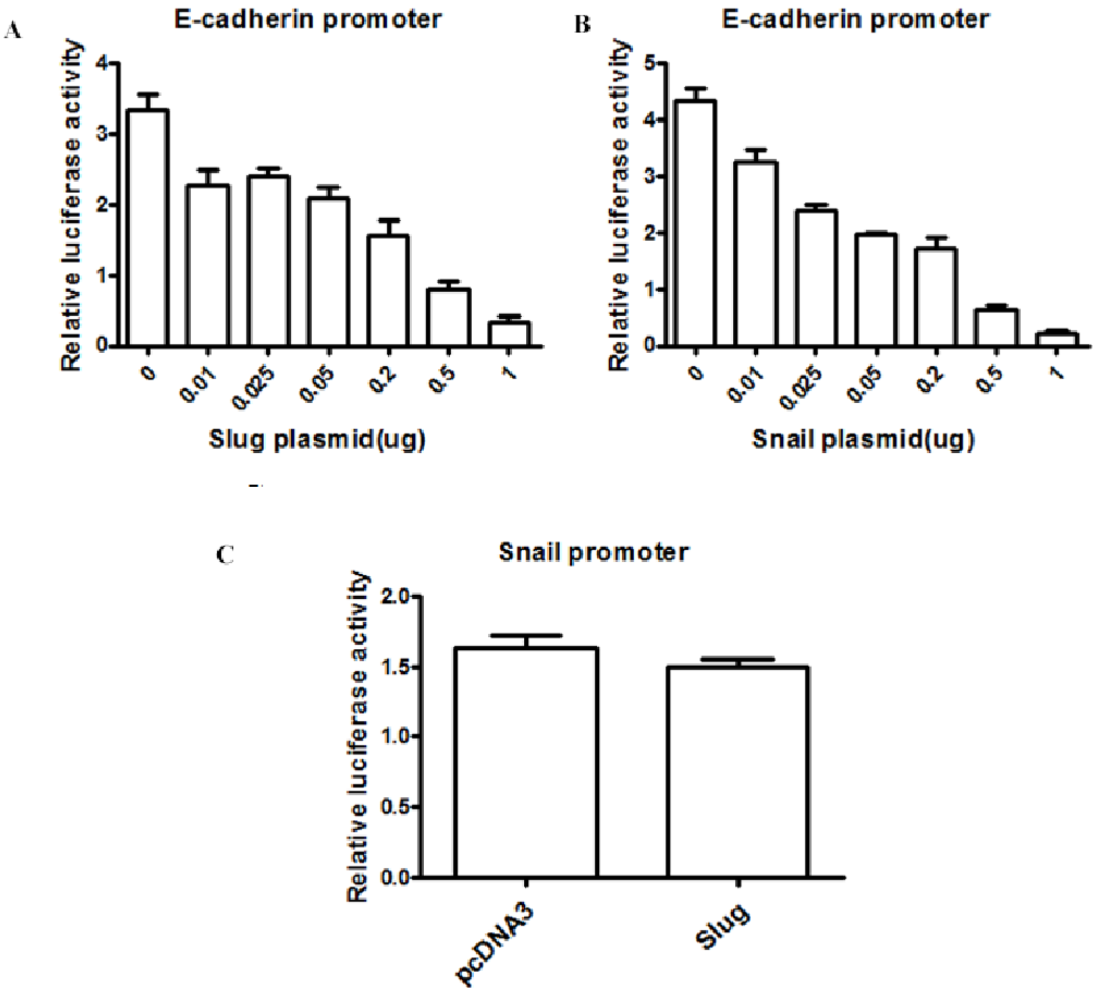

Supplementary Figure S3: The independent expression and regulation of Snail and Slug in cells. **(A)** 293T cells were transiently co-transfected with the indicated amounts of E-cadherin expression vectors (1 µg of the empty vector was used in column 1) and Slug promoter reporter. Relative luciferase activity is shown. Error bars represent mean ± SD of triplicate experiments. **(B)** 293T cells were transiently co-transfected with the indicated amounts of E-cadherin expression vectors (1 µg of the empty vector was used in column 1) and Snail promoter reporter. Relative luciferase activity is shown. Error bars represent mean ± SD of triplicate experiments. **(C)** 293T cells were transiently co-transfected with 1 µg of Slug expression construct or 1 µg of pcDNA3, together with Snail promoter. Relative luciferase activity is shown. Error bars represent mean ± SD of triplicate experiments.

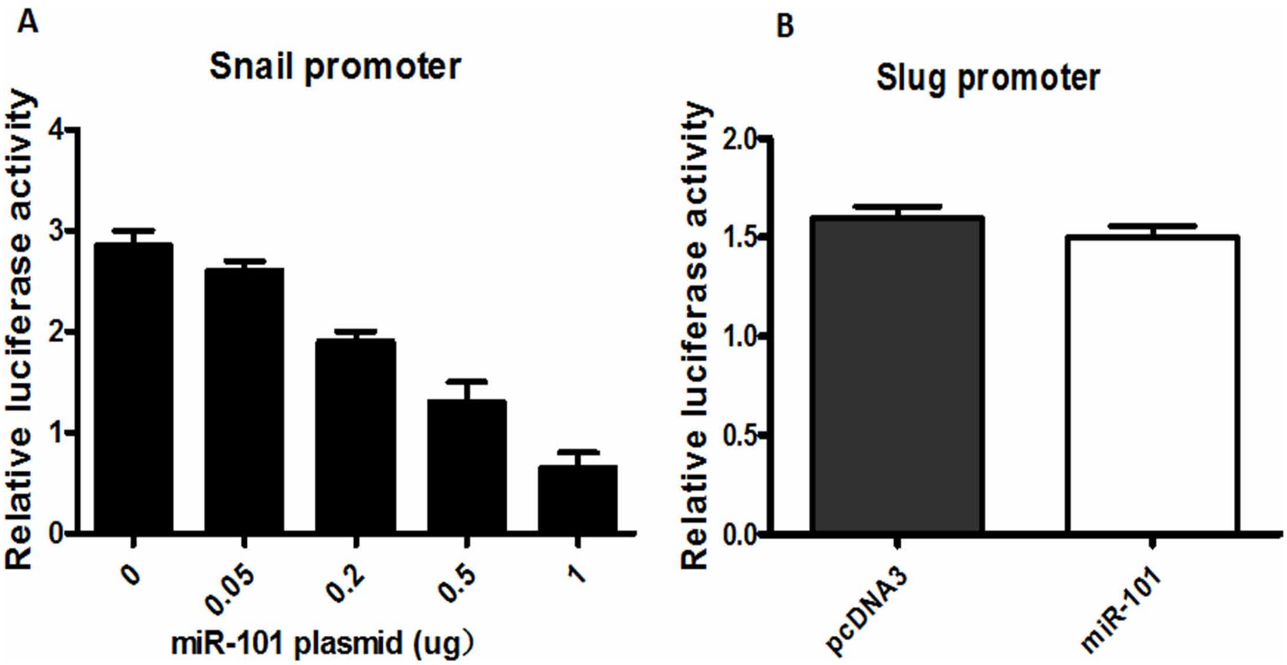

Supplementary Figure S4: miR-101 may directly regulate Snail transcription and indirectly regulate Slug transcription. **(A)** 293T cells were transiently co-transfected with the indicated amounts of Snail expression vectors (1 μg of the empty vector was used in column 1) and miR-101 promoter reporter. Relative luciferase activity is shown. Error bars represent mean ± SD of triplicate experiments. **(B)** 293T cells were transiently co-transfected with 1 μg of miR-101 expression construct or 1 μg of pcDNA3, together with Slug promoter. Relative luciferase activity is shown. Error bars represent mean ± SD of triplicate experiments.

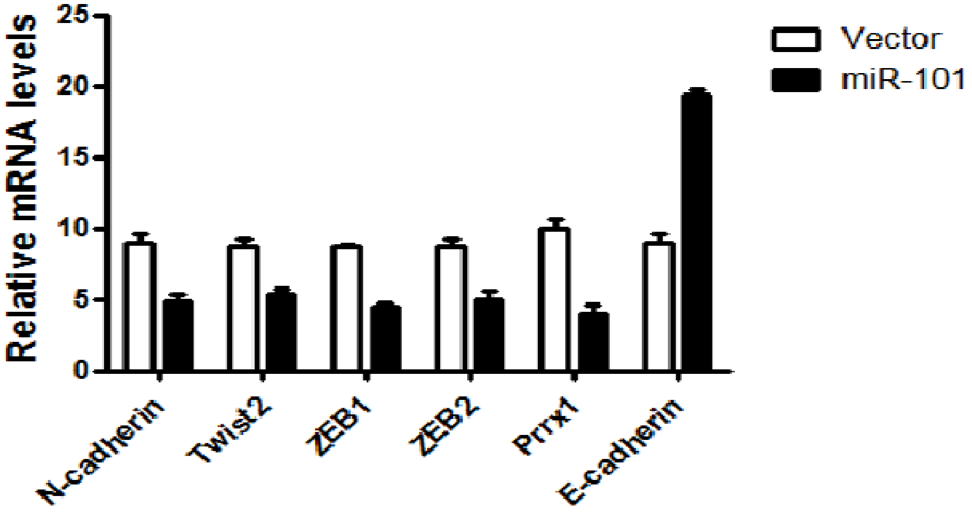

Supplementary Figure S5: The mRNA expression levels of known EMT inducers were assessed by real-time PCR. The mRNA levels of N-cadherin, Twist2, ZEB1, ZEB2, Prrx1 were reduced in response to miR-101 overexpression, whereas E-cadherin mRNA level was increased. Error bars represent the mean ± SD of triplicate experiments

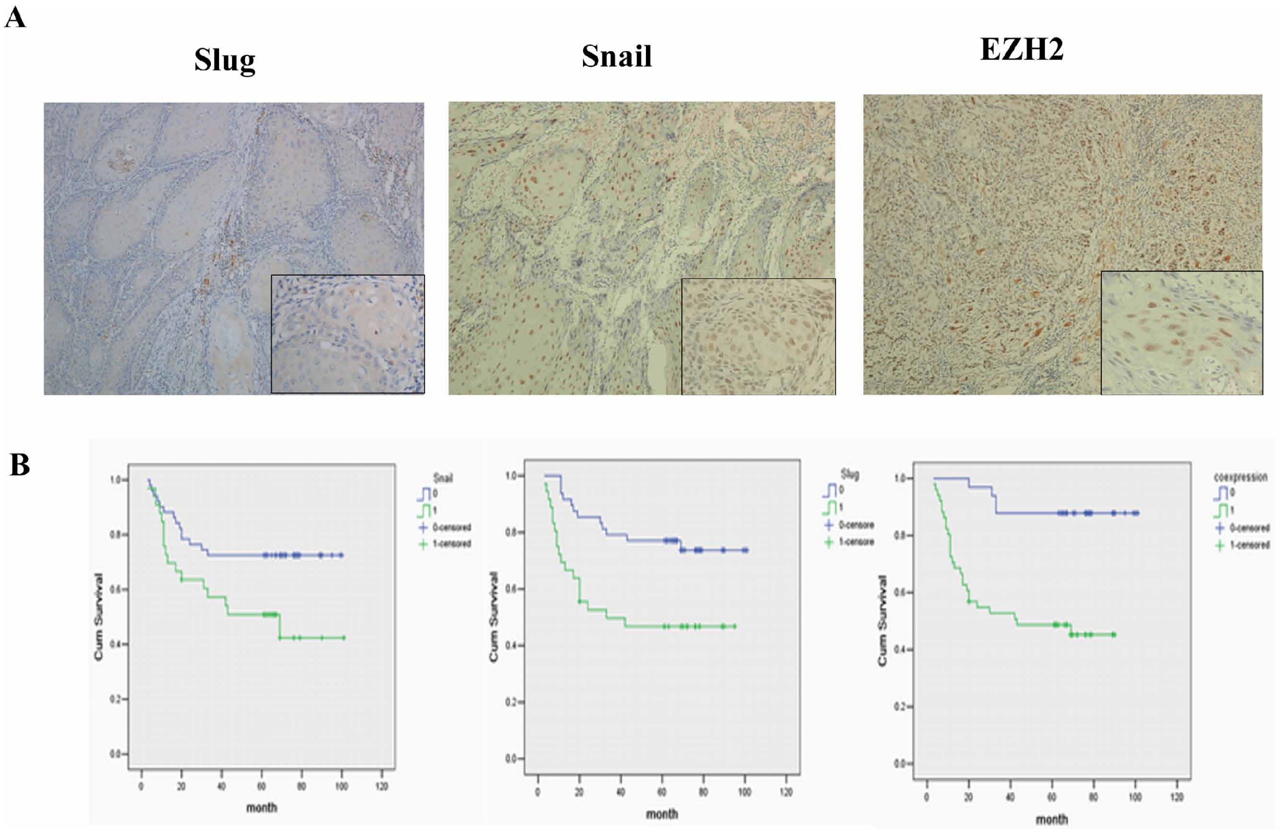

Supplementary Figure S6: Snail and Slug expression were associated with the poor prognosis of human OTSCC samples in Zhoushan. (A) Rrepresentative tissue sections stained for Snail, Slug and EZH2 by immunohistochemistry in OTSCC samples. Scale bar, 50 mm. **(B)** Kaplan-Meier survival analysis in patients with OTSCC. Co-overexpression of Snail and Slug in OTSCC was associated with a shorter overall survival rate, compared with the group with either of Snail and Slug overexpression.

Supplementary Table S1: Univariable survival analysis of clinical and pathological data of 89 oral tongue squamous cell carcinoma (OTSCC) patients

Clinicopathological features		No. of patients	Overall survival		Disease-free survival	
			Hazard ratio (95% CI)	*P* value	Hazard ratio (95% CI)	*P* value
Gender	Female	24	1.259(0.690–2.296)	0.453	1.080(0.644–1.828)	0.759
	Male	65				
Age	≤60	53	0.605(0.321–1.142)	0.121	0.778(0.456–1.328)	0.357
	> 60	36				
T classification	T1/T2	51	2.928(1.590–5.392)	0.001	3.987(2.294–6.929)	< 0.001
	T3/T4	38				
Pathological grade	Well	54	1.538(1.011–2.339)	0.044	1.633(1.125–2.369)	0.010
	Moderate	26				
	Poorly	9				
Local regional recurrence	without	55	3.047(1.668–5.567)	< 0.001	5.131(2.913–9.039)	< 0.001
	with	34				
Lymph node metastasis	without	52	1.952(1.079–3.533)	0.027	2.273(1.348–3.833)	0.002
	with	37				
Snail expression	Negative	53	2.021(1.021–3.245)	0.031	3.012(1.238–4.098)	0.024
	Positive	36				
Slug expression	Negative	39	2.201(1.450–3.308)	0.043	3.470(2.010–4.216)	0.035
	Positive	50				
Snail and Slug co-overexpression	Both	30	2.736(1.474–5.076)	0.001	3.268(1.888–5.657)	< 0.001
	Others	59				

Supplementary Table S2: Independent significant prognostic factors after Cox multivariate survival analysis of oral tongue squamous cell carcinoma (OTSCC)

Variable	Overall survival		Disease-free survival	
	Relative risk (95% CI)	*P* value	Relative risk (95% CI)	*P* value
Local recurrence	2.482 (1.330–4.630)	0.004	3.700 (2.055–6.664)	< 0.001
Lymph node metastasis			1.981(1.146–3.425)	0.014
Snail and Slug co-overexpression	2.463(1.243–4.504)	0.012	3.342(1.351–4.352)	< 0.001

Supplementary Table S3: Association between expression of E-cadherin, N-cadherin, Vimentin, and Snail+/Slug+ overexpression in patients with OTSCC

Snail+/Slug+ expression	E-cadherin expression		*P* value	N-cadherin expression		*P* value	Vimentin expression		*P* value
	No (*n* = 58)	Yes (*n* = 31)		No (*n* = 35)	Yes (*n* = 54)		No (*n* = 33)	Yes (*n* = 56)	
			0.009			0.004			0.002
No (*n* = 59)	34	25		30	29		29	30	
Yes (*n* = 30)	24	6		5	25		4	26	

Supplementary Table S4: Clinicopathological features of OTSCC patients and their association with Snail, Slug and EZH2 expression in another independent OTSCC patient cohorts (*n* = 40)

Clinicopathological features	*n*	Snail/Slug co-expression		*P* Value	EZH2 expression		*P* Value
		Both positive (*n* = 14)	Others (*n* = 26)		Positive (*n* = 23)	Negative (*n* = 17)	
**Age**	40			1.000			0.3322
< = 60	24	8	16		12	12	
> 60	16	6	10		11	5	
**Gender**	40			0.5103			0.1159
Female	18	5	13		13	5	
Male	22	9	13		10	12	
**T classification**	40			0.0408			0.0002
T1/T2	24	5	19		8	16	
T3/T4	16	9	7		15	1	
**Pathological grade**	40			< 0.001			0.0028
Well	24	2	22		9	15	
Moderate +Poorly	16	12	4		14	2	
**Lymph node metastasis**	40			0.0090			0.0011
Yes	17	10	7		15	2	
No	23	4	19		8	15	
**Local regional recurrence**	40			0.0168			0.0072
Yes	15	9	6		13	2	
No	25	5	20		10	15	
